# From classroom to screen: dental students’ perceptions of distance learning during COVID-19 pandemic in India

**DOI:** 10.1186/s12909-025-07906-0

**Published:** 2025-10-02

**Authors:** Shaswata Karmakar, Mehuli Kar, Delfin Lovelina Francis, Saravanan Sampoornam Pape Reddy, Baishakhi Modak

**Affiliations:** 1 Department of Periodontology, Manipal College of Dental Sciences Manipal, Manipal Academy of Higher Education, Manipal, India; 2 Department of Public Health Dentistry, Saveetha Dental College and Hospitals, Saveetha University, Saveetha Institute of Medical and Technical Sciences, Chennai, India; 3 Department of Periodontology, Army Dental Corps, New Delhi, India; 4 Department of Pharmacy Practice, Manipal College of Pharmaceutical Sciences Manipal, Manipal Academy of Higher Education, Manipal, India

**Keywords:** Distance Learning, Distance Education, Dentae Education, COVID-19, Medical Education, Technology, Education Model

## Abstract

**Background:**

The COVID-19 pandemic had severely disrupted the education system in a variety of settings, particularly medical and dental teaching institutes. Restricting the teaching system to virtual mode and the treatment aspect to emergency-only care was of great challenge, in order to prevent the spread of COVID-19. During the pandemic, distance learning had become necessary to ensure that education continues.

**Objective:**

The present study aimed to analyse dental students’ perceptions of the transition of education from traditional to distance learning.

**Methods:**

This was a cross-sectional study consisting of a 25-statement online questionnaire, which was validated (Item- Content Validity Index (I-CVI: 0.87–0.89), reliability confirmed (Cronbach’s alpha = 0.82). The questionnaire was anonymously administered to students studying dentistry at the Manipal Academy of Higher Education, Manipal, India. The data collected were analysed statistically.

**Results:**

A total of 713 out of 800 undergraduate dental students participated in the survey, resulting in an 89.12% response rate. Approximately 74% students did not feel that it was easier to concentrate in online classes than in offline lectures, and 60.9% of the students felt that they could not learn the theoretical aspects adequately. Almost three-fourths of the students (73.9%) felt that lockdowns and distance education severely affected the quality of their dental education. Most of the students (86.4%) felt that a lack of patient exposure would affect their future dental practice. With respect to learning preferences, the majority of the students preferred a combination of traditional methods and online learning.

**Conclusion:**

The COVID-19 pandemic taught not only the use of technology in education but also future learning strategies. In today’s technologically adept world, e-learning is a convenient and effective method for teaching undergraduate dental students. Dental education is evolving consistently to accommodate rapid changes in the education system. However, it should be used as an auxiliary approach in the clinical setting since it cannot replace the conventional face-to-face approach.

**Supplementary Information:**

The online version contains supplementary material available at 10.1186/s12909-025-07906-0.

## Introduction

COVID-19, also known as coronavirus disease 2019 (COVID-19), had been recognised as a viral infection caused by severe acute respiratory syndrome coronavirus-2 (SARS-CoV-2), which created a public health crisis worldwide in late 2019 [1]. Several measures had been taken to minimise the spread of contagion, including isolation, contact tracing, quarantine, social and physical distancing, hygiene measures and lockdown. Among these measures, social distancing had been the most widely used measure taken to protect people from the spread of the virus [1]. The education system, being the backbone of a society as well as a country, was among the sectors that were severely affected by the pandemic. Over 1.5 billion students globally (90.1% of total enrolled students) were impacted by educational changes during the COVID-19 pandemic, according to UNESCO [2]. Medical education, specifically dental education, was no exception from being affected. Since social distancing was the need of the hour, the offline face-to-face classroom education system was interrupted worldwide. Learning in dentistry involves the communication and treatment of patients through the oral cavity, which is a reservoir for the COVID-19 virus. This exposes dental professionals to saliva, blood, and several other body fluids, which puts them at high risk of becoming infected as well as a source of viral spread to others [3]. Moreover, droplet- and aerosol-generating dental treatment procedures, such as tooth preparation, ultrasonic scaling, and cavity preparation, are considered among the most effective modes of transmission of the virus in a dental clinical setting [4, 5]. Therefore, restricting the teaching system to the virtual mode and the treatment aspect to emergency-only care seemed to be a realistic choice to prevent the spread of COVID-19 [5]. Although online education is not a very new concept for educators in general, it had emerged as a global need during the COVID-19 pandemic. Virtual learning can take the form of online theory and practical classes, assignments, and assessments. The rapid development of online teaching platforms and smart devices such as mobile devices, laptops, and tablets had provided convenient ways for students to attend classes and contact teachers [6]. Students could continue to participate in live academic lectures in the web-based virtual environment, and they could also access these lectures whenever and wherever they needed them later. Virtual patients also aided dental students in developing clinical skills such as obtaining case histories, observing signs and symptoms, etc [7]. Various tools, such as PowerPoint presentations, live or recorded lectures, video demonstrations, case scenarios, online whiteboards, and virtual models, had been used [8, 9]. However, dentistry is a “professional artistry” by nature, as described by Robert Caplin et al. [10]. The success of a dental treatment depends largely on the degree of skill the operator possesses. This “professional skill” is a combination of ability, expertise and cleverness, which dictates the ultimate outcome of a dental treatment [10]. Therefore, virtual methods of learning are of limited use in dental education, even though timely dissemination of information about COVID-19 by the Ministry of Health and the government has had a significant effect on knowledge and awareness [11, 12]. Amir et al. evaluated the perspective of distance learning compared with that of classroom learning and concluded that most of the students preferred learning that combines both classroom and online modes [13]. However, contradictory results have also been reported in several studies. Sarialioglu et al. evaluated the perceptions and attitudes of dental students toward online distance learning and concluded that dental students were dissatisfied with the disruption of traditional patterns of education [14]. The results from another study conducted by Di Giacomo et al. revealed that most of the students were not satisfied with online distance learning, especially the preclinical and clinical aspects of the curriculum [15]. India, being a densely populated, developing country with almost 1.5 billion people living, the impact of COVID-19 infection was of greater magnitude compared to the other parts of the world. Moreover, limited infrastructure and sudden pedagogical changes posed unique challenges. Hence, preventing the spread of the virus by switching the mode of education from conventional face-to-face to distance learning was of utmost importance. Shrivastave et al. conducted a nation-wide descriptive survey to understand the pros and cons of online education among undergraduate dental students across India. The study concluded that the education of dental students was adversely affected as a result of COVID-19 pandemic. The physical and psychological well-being of the students had also been severely affected [16]. However, literature investigating the impact of COVID-19 and the perspectives and attitudes of dental students towards sudden changes in the education system due to the pandemic, considering the Indian scenario, is very limited. Also, in order to better understand student’s acceptance and motivation towards distance learning, the present study incorporates the Technology Acceptance Model (TAM). TAM suggests that perceived usefulness (PU) and perceived ease of use (PEOU) are primary factors influencing user’s attitudes and intentions to adopt technology [17]. Integrating this conceptual lens helps interpret student’s perceptions of the shift to virtual education in the Indian dental context. Therefore, the aim of our study was to evaluate the perspective of dental students at an Indian university towards distance learning.

## Materials and methods

The study was a cross-sectional survey. Undergraduate students studying professional Bachelor of Dental Surgery (B.D.S.) course at Manipal Academy of Higher Education, Manipal, India, who agreed to participate in the study and gave informed consent electronically were included in the study. Students who were not willing to participate were excluded from the study. The study was conducted from July to December 2021. Students were informed about the study, and e-signed consent was obtained from them. This study was reported in compliance with the Strengthening the Reporting of Observational Studies in Epidemiology (STROBE) statement. The study protocol was in accordance with the Helsinki Declaration and was reviewed and approved by the Institutional Ethical Committee (IEC no. 370/2021, Kasturba Hospital Institutional Ethical Committee, Manipal). The questionnaire was created via Google forms (Google LLC, California, USA), and was distributed via institutional e-mail and WhatsApp.

### Validity and reliability of the questionnaire

The Davis criteria for the content validity index were used to validate the questionnaire before starting the study. The questionnaire was given to two healthcare experts. The item content validity index (I-CVI) scores for the experts were 0.87 and 0.89, respectively. For reliability testing, five undergraduate students from each professional year completed the questionnaire for verification. The correlation coefficient (Cronbach’s alpha) was 0.82, indicating a good degree of agreement.

All eligible students were invited to participate in a voluntary survey. The sample size required for the present study was calculated using the standard formula for sample size calculation. Considering potential errors and sample loss, which is common in cross-sectional studies, a final sample size was estimated to be 650 based on a confidence interval of 95% and a margin of error of 5% with an estimated response rate of 60% using a statistical software. In order to pick the study subjects from our sampling frame, a census-based sampling approach was used, inviting all undergraduate dental students enrolled at the institution during the study period to participate. The questionnaire was administered anonymously, reducing social desirability bias. The use of a structured, pretested questionnaire helped limit interpretation variability. Additionally, the inclusion of all the students studying B.D.S., regardless of the year, ensured diverse perspectives. These factors could effectively control possible selection and response bias. The questionnaire consists of 25 questions that include 5 sections (demographic details, acceptance, didactic learning, preclinical and clinical learning, and motivation). The response options for the questionnaire items represent a combination of ‘yes’ or ‘no’ responses and Likert-type scales (strongly disagree to strongly agree) (Annexure 1).

### Statistical analysis

Descriptive statistics were computed, and analysis was performed via SPSS software version 28.0 (IBM LLC, Armonk, New York).

## Results

### Demographic data

**Fig. 1:**
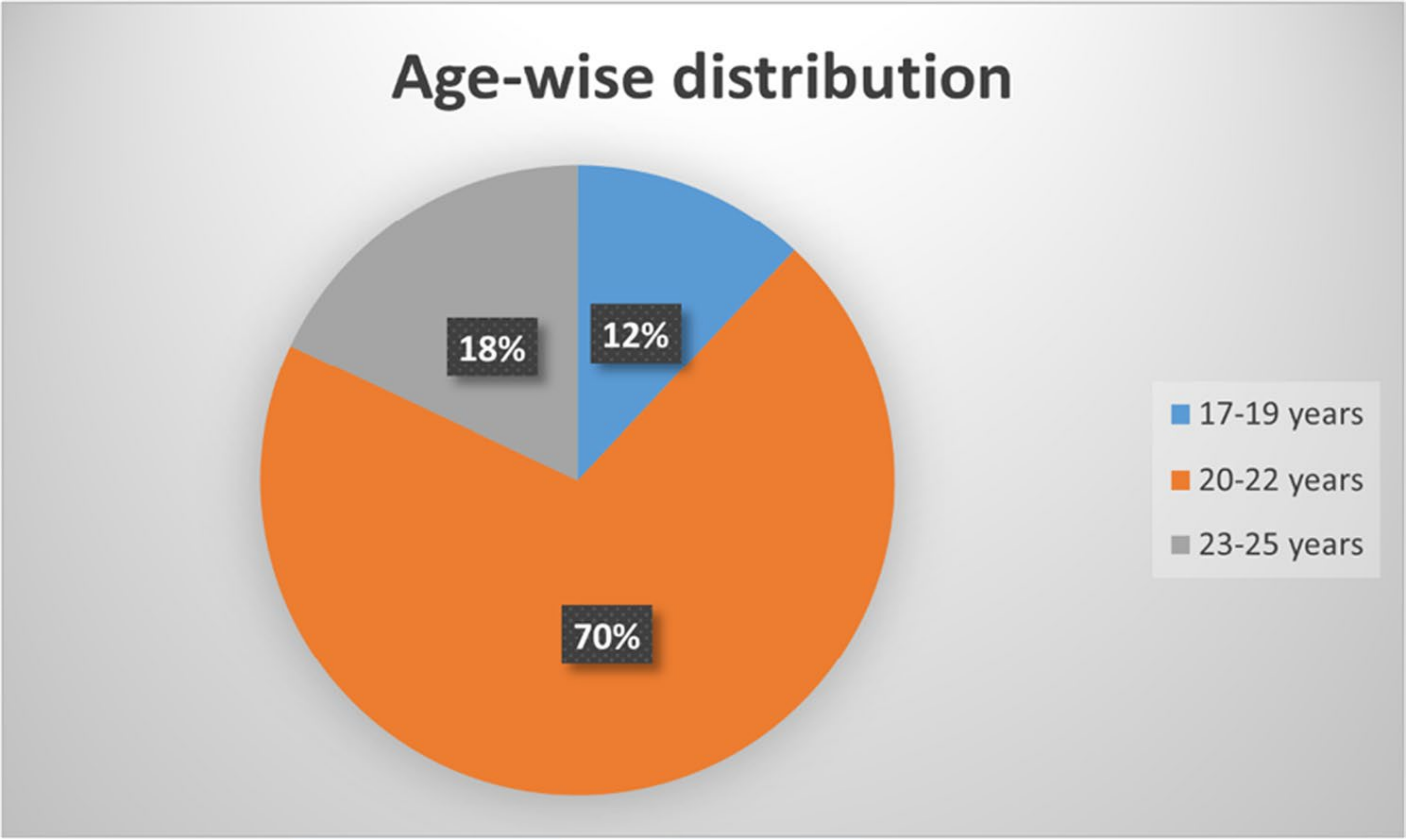
Age-wise frequency distribution of the study participants

**Fig. 2:**
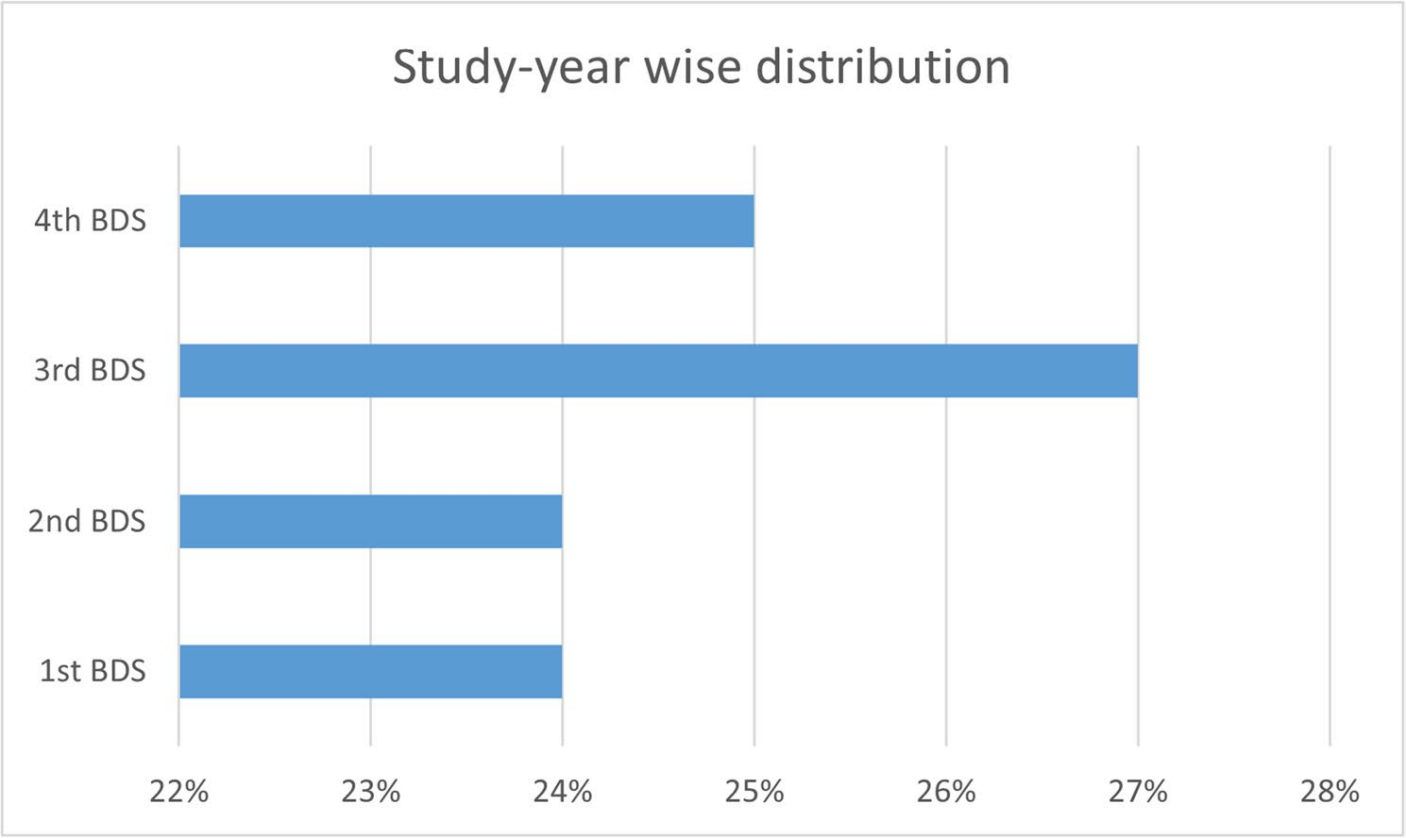
Study year wise frequency distribution of the study participants

**Fig. 3:**
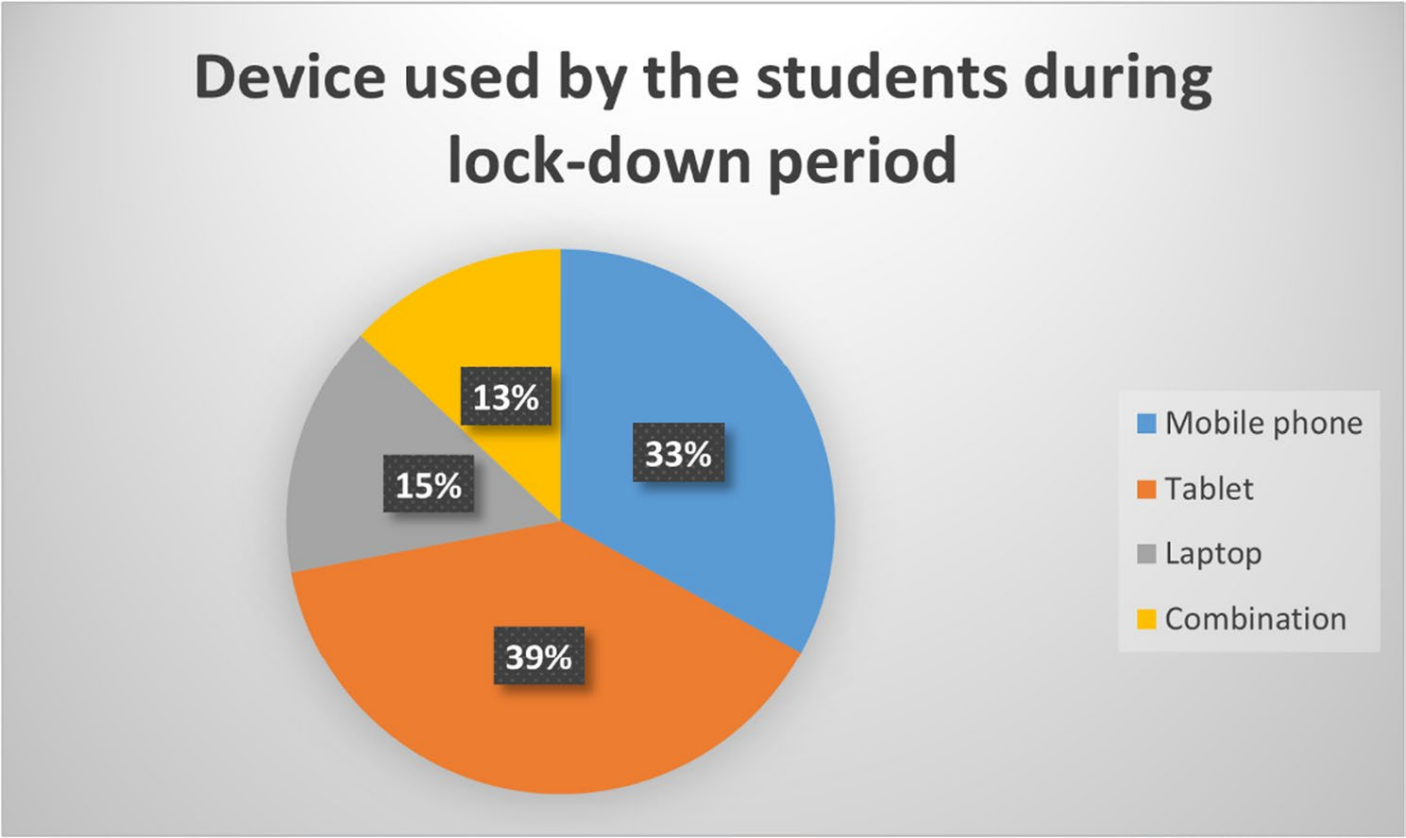
Frequency distribution of the devices used by the participants to attend online classes during the lockdown period

Out of 800 undergraduate students who were studying dentistry at MAHE, Manipal, a total of 713 participants, comprising students in the 1st- to 4th-year professional B.D.S., responded to and completed the questionnaire. This represented a response rate of 89.12%. The age of the students ranged from 17 to 25 years. The age wise and study year wise frequency distributions are presented in Figs. 1 and 2. The students used mobile phones, laptops, tablets, or their combination to attend online classes (Fig. 3).

#### Acceptance

The majority of the students (74.2%) had not been exposed to distance learning before this pandemic. They used laptops, mobile devices, tablets, or combinations of these to attend classes. A total of 41.9% of the students felt that they were well prepared for distance learning methods. One-fourth of the students (27.3%) did not have access to sufficient internet facilities. However, both online class timings and the surrounding environment were suitable for the majority of the students (84.3% and 73.2%, respectively) (Table 1).

**Table 1:** Acceptance

Questions	Options	F value	%
1. Apart from Covid-19 era, have you been exposed to distance e-learning before?	No	529	74.2
	Yes	184	25.8
2. Were you well prepared in advance for the distance e-learning methods?	No	414	58.1
	Yes	299	41.9
3. Were distance e-learning session timings suitable for you?	No	112	15.7
	Yes	601	84.3
4. Did you have access to sufficient internet facility to participate in online classes?	No	195	27.3
	Yes	518	72.7
5. Did you have a suitable environment around while you attended online classes?	No	191	26.8
	Yes	522	73.2

In terms of didactic learning, 73.9% of the students did not feel it was easier to concentrate in online classes than in offline lecture sessions, and 60.9% of the students felt that they could not learn the theoretical aspects adequately. More than half (57.4%) of the students reported that attending online classes was stressful. However, almost two-thirds of the students (69.3%) felt that they had ample opportunity to clear their doubts post e-lectures (Table 2).

**Table 2:** Didactic learning

Questions	Options	f value	%
1. Online classes are stressful	No	304	42.6
	Yes	409	57.4
3. Do you feel easier to concentrate in online classes as compared to offline lecture sessions?	No	527	73.9
	Yes	186	26.1
5. Do you feel you have learnt the theoretical concepts adequately through distance e-learning?	No	434	60.9
	Yes	279	39.1
6. Do you feel online e-learning was a good option for understanding the theoretical part of your curriculum?	No	413	57.9
	Yes	300	42.1
7. Was there ample opportunity to clear your doubts during or post e-lectures?	No	219	30.7
	Yes	494	69.3
8. At the time of online learning, did you feel that you could ask questions regarding the topic or subject being taught, more often compared to that during classroom lectures?	No	387	54.3
	Yes	326	45.7

### Preclinical and clinical learning

In total, 73.9% and 65.9% of the students had preclinical and clinical components in their curriculum, respectively. The majority of the students felt that they could not grasp the concepts adequately (74.6% and 82% for preclinical and clinical aspects, respectively). A total of 83.7% of the students felt that the case-based scenarios, demonstrations and resources used for teaching preclinical and clinical portions were not as effective as handling live patients (Table 3).

**Table 3:** Preclinical and clinical learning

Questions	Option	f value	%
1. Did you have preclinical component(s) in your curriculum?	No	186	26.1
	Yes	527	73.9
2. If yes, do you feel you have grabbed the preclinical concepts and practices adequately through distance e-learning sessions?	No	532	74.6
	Yes	181	25.4
3. Did you have clinical component(s) in your curriculum?	No	243	34.1
	Yes	470	65.9
4. If yes, do you feel you have understood the clinical concepts and practices adequately through distance e-learning sessions?	No	585	82
	Yes	128	18
5. Do you feel the resources/demonstrations for teaching clinical/preclinical portions of the syllabus during e-learning process to be equally effective as handling the live patient?	No	597	83.7
	Yes	116	16.3

### Motivation

**Fig. 4:**
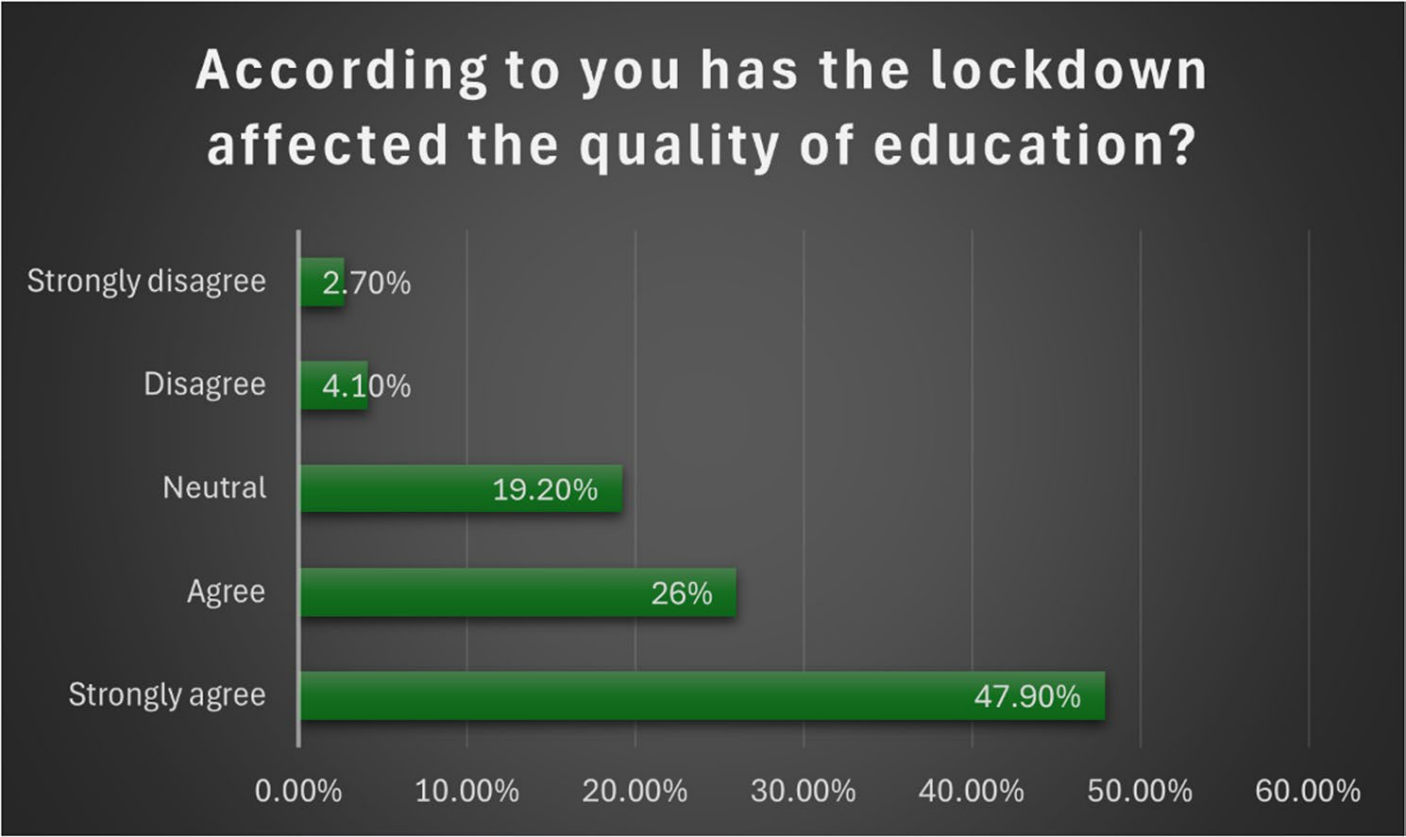
Participants’ agreement with the quality of education during the COVID-19 lockdown period

A total of 47.9% of the students strongly agreed that the lockdown severely affected their quality of education (Fig. 4). A total of 86.4% of the students felt that a lack of patient exposure would affect their future dental practice. A total of 61.4% of the students said that the newly implemented distance education system did not motivate them to learn more; therefore, they did not want it to continue after the COVID-19 pandemic (65.8%) (Table 4).

**Table 4:** Motivation

Question	Option	f value	%
1. Did you find the case-based scenarios used during online teaching beneficial to develop clinical skills?	No	415	58.2
	Yes	298	41.8
2. Do you think lack of patient exposure can affect your future dental practice?	No	97	13.6
	Yes	616	86.4
3. Does the implementation of the new distance education method motivate you to learn more?	No	438	61.4
	Yes	275	38.6
4. Do you wish distance e-learning sessions to continue post Covid-19 period instead of offline equivalents?	No	483	65.8
	Yes	230	34.2

## Discussion

This cross-sectional study was conducted to evaluate dental students’ perceptions of and experience with the distance education system, which was implemented by MAHE Manipal. Before the pandemic, the teaching method used in dentistry at MAHE was student-centered active blended learning. The teaching of theoretical aspects of the curriculum involves taking lecture classes face-to-face in the classroom via PowerPoint presentations, chalk and board presentations or a combination of them. Impartus web-based education tools (Impartus Innovations Private Limited, Bengaluru, India) were used to capture various lecture-based activities. Through this, students could access the lecture class, questions, learning objectives and syllabus of each course studied. For both preclinical and clinical aspects, question-based learning, outcome-based learning and collaborative learning approaches were used. For the clinical postings, the students were divided into small groups and were mentored by a subject teacher. Under the supervision of teaching faculties, students could learn various clinical skills by examining, diagnosing and treating patients who reported to the out-patient department. Overall, the teaching system represented blended learning. Since the pandemic protocol mandated social distancing and work-from-home patterns in March 2020, the teaching system has shifted completely to virtual mode. Lecture classes, doubt-clearing sessions, group discussions, and assessments were carried out via an online platform (Microsoft Teams, Microsoft LLC, USA). Preclinical and clinical portions of the syllabus in which psychomotor skills were required were substituted with video demonstrations and online simulation exercises. The first step taken by many dental educational institutions during the COVID-19 pandemic was to completely shut down or restrict education and service delivery to emergencies only. When it was anticipated that the pandemic would last for a much longer period, the institutions started to implement new and different strategies to continue their education, research, and treatment activities with the lowest possible risk of infection for their students, patients, and staffs. On the 11th of March, 2020, the World Health Organisation (WHO) declared COVID-19 a pandemic. Originating in Wuhan Province in China, COVID-19 has spread to more than 200 countries worldwide [18]. As of December 2021, more than 90 million people have been affected, with more than 1.8 million deaths, and this number is kept increasing each day [19]. COVID-19 is a highly transmissible disease. The United States Occupational Safety and Health Administration (US-OSHA) categorised dental treatments as very high risk due to several factors, such as aerosol generation and exposure to saliva and blood [13]. Avoidance of crowding and physical distancing was a challenge to minimise the spread of the infection, especially in a densely populated country like India. During the pandemic, the greatest challenge for teachers in dental colleges was to decrease the spread of COVID-19 infection without the interruption of dental education. Distant learning methods have therefore been adopted by almost all universities worldwide [20].

**Fig. 5:**
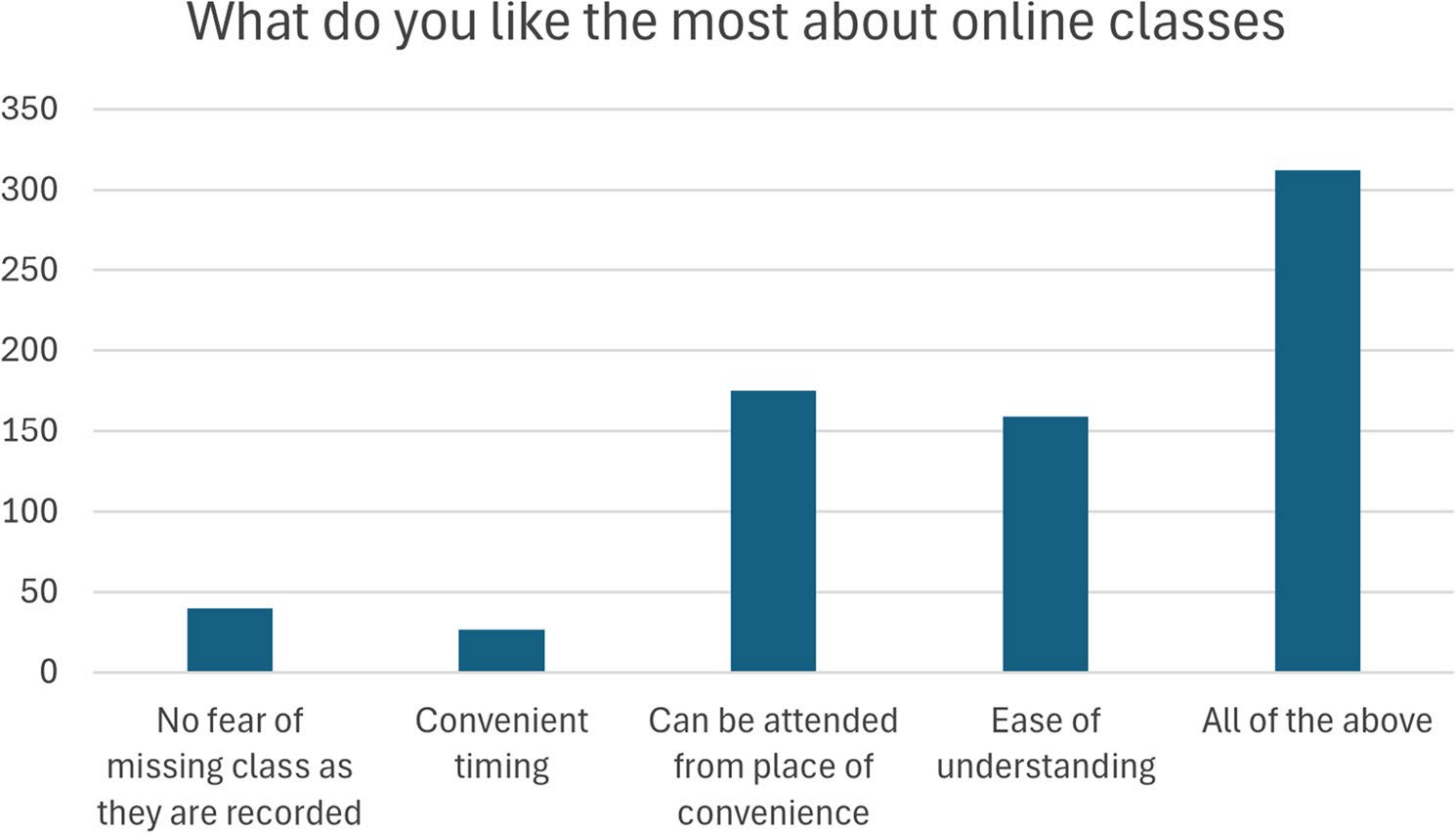
Response of the participants regarding the most preferred fact about distance learning

During this period, MAHE Manipal used various learning methods, such as lecture-based education (LBE), group learning (GL), problem-based education (PBE), and case-scenario-based learning (CSL). LBE focuses mainly on didactic learning, which consists of factual knowledge delivery on a particular topic. In GL, the students are divided into small groups, topics are discussed, assignments are given, and online tests are performed. PBE is the application of theoretical knowledge for the solution of practical problems. This approach was used mainly for preclinical exercises. The CSL was prepared to simulate clinical cases. In this method, students are given the history and other clinical findings of a patient. The students were then asked to apply their basic knowledge of the disease and assess the scenario to accurately diagnose the condition and plan a line of treatment. In the present study, concerning the theoretical aspects of the curriculum, students expressed several advantages of online classes, such as convenient timing of the classes, convenience of attending classes from any place, ease of understanding, and no fear of missing classes, as they are recorded and saved for future reference (Fig. 5). This, in turn, reduced the necessity of conducting revision classes on very important topics. The majority of the students also agreed that they had ample opportunity to clear their doubts after the lecture session and that they could ask questions regarding the topic taught more often than they had during classroom lectures. However, almost two-thirds of the students felt a lack of concentration in online classes compared with offline face-to-face lectures. This might be due to the lack of an appropriate environment for studying at a location that is distant from the teaching institute. In a review, Dhawan et al. discussed the drawbacks associated with online teaching and concluded that online teaching was not an alternative but rather a necessity during a pandemic [21]. Distance learning was more challenging in terms of the preclinical and clinical aspects of the curriculum. Despite these drawbacks, enormous efforts have been made to overcome these challenges. Several online tutorial programs were organised by the university to guide faculty members in various online activities. E-modules were used to teach the students about practicing hands-on and various stimulating exercises for their clinical skills at home. However, the majority of the students felt that the preclinical and clinical aspects of the curriculum could be learned better via a face-to-face learning approach than via a distant approach. Dentistry is an occupation whose core element is work based upon the mastery of a complex body of knowledge and skills [22]. The efficacy of dental treatments depends on the skill of the operator, which can be defined as practical knowledge in combination with ability, cleverness and expertise [22]. These skills could not be completely learned without exposure to preclinical models and patients, which might be the reason that the students were dissatisfied with distance learning. Our findings are in line with those of several other studies [23, 24]. However, the results of our study revealed mixed perceptions and attitudes toward online education during the COVID-19 pandemic. The negative feedback by the students resulted from the mental stress created by several factors, such as suddenly staying at home for an extended period, not being able to meet their peers, lockdown, uncertainty about health and education, isolation, and back-to-back lecture classes [25]. To date, there is no gold-standard approach to address such a pandemic situation. All the teaching methods implemented were on a trial-and-error basis. Although many students were satisfied with distance learning, they presented a predominantly negative perspective on the implementation of complete shift to distance learning. The results of our present study showed that the face-to-face system was preferred by the students and that they did not want the distance learning system to continue for a long period of time. Their opinions, such as “lack of patient exposure would affect our future dental practice” and “implementation of the new distance learning method does not motivate us to learn more,” proved the same. Our results are consistent with those of a recent study performed by Noor et al., who concluded that e-learning can be accepted only partially and not as a single method of education in dentistry [26].

The experience of the COVID-19 pandemic has emphasised the importance of improving computer operating skills not only for students but also for teaching faculties. Special skills to communicate with students are essential since a lack of personal contact may affect communication between teachers and students. It is also mandatory to prepare in advance for similar crises that may occur in the future. Dental educators, researchers and dental professionals must create a system to address the new normal. Many efforts have been made to build an online lecture system, develop blended modules and virtual reality (VR) laboratories for online simulated training, develop an online exam system, etc. to maintain the quality of education [27].

Although many studies have explored dental student’s experiences with online learning during COVID-19 in developed countries, our study targeted undergraduate dental students at a prominent Indian university who represent a specific cohort with educational challenges such as overcrowded teaching environments, technological scarcity and changing teaching methods. The study uses a reliable, pretested questionnaire to gather detailed data on student’s acceptance, learning experience, and motivation, highlighting both educational and emotional impacts. The study findings can help shape improvements in technology on campus, how classes are taught and how emergency plans are drawn up. Also, the findings offer clear recommendations to enhance digital resources and teaching support for future disruptions in dental education. The findings of our study also align with the Technology Acceptance Model (TAM), as students with the higher perceived usefulness and ease of use are more likely to accept and use virtual education [17]. However, there were challenges such as inadequate internet and lack of patient exposure, demonstrate limitations in perceived usefulness and ease, ultimately reducing student’s intention to fully adopt online learning. The use of TAM therefore with aspects of technological transition require targeted interventions in order to enhance their educational effectiveness and acceptance. There were several limitations of our study. The survey was performed in a single dental school in India, with the participation of 713 dental students. To view the effects of an unprecedented situation such as COVID-19 on the dental education system, more participants from more dental schools are needed for further study. Additionally, this study was a cross-sectional survey. Longitudinal studies are needed to understand the efficacy of the implemented teaching and learning strategies. Additionally, our study did not include students who were doing their internship. The effect of distance learning on students in the internship stage needs further research.

Therefore, with all the technological advancements, a combination of physical and online classes in terms of blended learning courses will be the future trend for dental education, i.e., in-person and clinical training should be combined with virtual education [28]. However, currently, no teaching method can simulate real patient and diagnosis and treatment processes exactly, and more informative projects need to be developed in the future. The results of our survey can help implement strategies to manage an unprecedented crisis and minimise its impact on the dental education system in the future. This would also help institutions be better prepared for similar disruptions. However, the most important thing is ensuring the quality of education.

## Conclusion

Within the limitations of the study, it could be concluded that distance learning can only be an adjunct to conventional methods, especially in dentistry. Blended learning via hybrid methods, i.e., the use of both virtual platforms and face-to-face teaching, is the method of choice for dental education to ensure the quality of education and to be updated with technological advances.

## Supplementary Information

Supplementary Material 1.

## Data Availability

The datasets used and/or analysed during the current study are available from the corresponding author upon reasonable request.
