# Supplementary material for: From classroom to screen: dental students’ perceptions of distance learning during COVID-19 pandemic in India
Source: BMC Med Educ. 2025 Oct 2;25:1334. doi: 10.1186/s12909-025-07906-0 (PMC12492926; doi:10.1186/s12909-025-07906-0)
Supplement: Supplementary file 1 — Supplementary Material 1. [file 12909_2025_7906_MOESM1_ESM.docx]

**Annexure 1: Questionnaire**

| **Demographic details Questions** | **Options** |
| --- | --- |
| 1. Age | 17-19 |
|  | 20-22 |
|  | 23-25 |
| 1. Year of study | First year |
|  | Second year |
|  | Third year |
|  | Fourth year |
| 1. what device did you use during lockdown period ? | Mobile |
|  | Laptop |
|  | Tablet |
|  | Combination |
| **Acceptance Questions** |  |
| 1. Apart from Covid-19 era, have you been exposed to distance e-learning before? | No |
|  | Yes |
| 1. Were you well prepared in advance for the distance e-learning methods? | No |
|  | Yes |
| 1. Were distance e-learning session timings suitable for you? | No |
|  | Yes |
| 1. Did you have access to sufficient internet facility to participate in online classes? | No |
|  | Yes |
| 1. Did you have a suitable environment around while you attended online classes? | No |
|  | Yes |
| **Didactic Learning Questions** | **Options** |
| 1. Online classes are stressful | No |
|  | Yes |
| 1. Do you feel easier to concentrate in online classes as compared to offline lecture sessions? | No |
|  | Yes |
| 1. Do you feel you have learnt the theoretical concepts adequately through distance e-learning? | No |
|  | Yes |
| 1. Do you feel online e-learning was a good option for understanding the theoretical part of your curriculum? | No |
|  | Yes |
| 1. Was there ample opportunity to clear your doubts during or post e-lectures? | No |
|  | Yes |
| 1. At the time of online learning, did you feel that you could ask questions regarding the topic or subject being taught, more often compared to that during classroom lectures? | No |
|  | Yes |
| **Preclinical and Clinical Learning Questions** | **Option** |
| 1. Did you have preclinical component(s) in your curriculum? | No |
|  | Yes |
| 1. If yes, do you feel you have grabbed the preclinical concepts and practices adequately through distance e-learning sessions? | No |
|  | Yes |
| 1. Did you have clinical component(s) in your curriculum? | No |
|  | Yes |
| 1. If yes, do you feel you have understood the clinical concepts and practices adequately through distance e-learning sessions? | No |
|  | Yes |
| 1. Do you feel the resources/demonstrations for teaching clinical/preclinical portions of the syllabus during e-learning process to be equally effective as handling the live patient? | No |
|  | Yes |
| **Motivation Questions** | **Options** |
| 1. Did you find the case-based scenarios used during online teaching beneficial to develop clinical skills? | No |
|  | Yes |
| 1. Do you think lack of patient exposure can affect your future dental practice? | No |
|  | Yes |
| 1. Does the implementation of the new distance education method motivate you to learn more? | No |
|  | Yes |
| 1. Do you wish distance e-learning sessions to continue post Covid-19 period instead of offline equivalents? | No |
|  | Yes |
| 1. According to you, has the lockdown affected the quality of education? | Strongly disagree |
|  | Disagree |
|  | Neutral |
|  | Agree |
|  | Strongly agree |
| 1. What do you like the most about online classes ? | No fear of missing class as they are recorded |
|  | Convenient timing |
|  | Can be attended from place of convenience |
|  | Ease of understanding |
|  | All of the above |
